# Cigarette Smoke Disturbs the Survival of CD8^+^ Tc/Tregs Partially through Muscarinic Receptors-Dependent Mechanisms in Chronic Obstructive Pulmonary Disease

**DOI:** 10.1371/journal.pone.0147232

**Published:** 2016-01-25

**Authors:** Gang Chen, Mei Zhou, Long Chen, Zhao-Ji Meng, Xian-Zhi Xiong, Hong-Ju Liu, Jian-Bao Xin, Jian-Chu Zhang

**Affiliations:** Department of Respiratory and Critical Care Medicine, Key Laboratory of Pulmonary Diseases of Health Ministry, Union Hospital, Tongji Medical College, Huazhong University of Science and Technology, Wuhan, Hubei, China; Georgia Regents University, UNITED STATES

## Abstract

**Background:**

CD8^+^ T cells (Cytotoxic T cells, Tc) are known to play a critical role in the pathogenesis of smoking related airway inflammation including chronic obstructive pulmonary disease (COPD). However, how cigarette smoke directly impacts systematic CD8^+^ T cell and regulatory T cell (Treg) subsets, especially by modulating muscarinic acetylcholine receptors (MRs), has yet to be well elucidated.

**Methods:**

Circulating CD8^+^ Tc/Tregs in healthy nonsmokers (n = 15), healthy smokers (n = 15) and COPD patients (n = 18) were evaluated by flow cytometry after incubating with anti-CD3, anti-CD8, anti-CD25, anti-Foxp3 antibodies. Peripheral blood T cells (PBT cells) from healthy nonsmokers were cultured in the presence of cigarette smoke extract (CSE) alone or combined with MRs agonist/antagonist for 5 days. Proliferation and apoptosis were evaluated by flow cytometry using Ki-67/Annexin-V antibodies to measure the effects of CSE on the survival of CD8^+^ Tc/Tregs.

**Results:**

While COPD patients have elevated circulating percentage of CD8^+^ T cells, healthy smokers have higher frequency of CD8^+^ Tregs. Elevated percentages of CD8^+^ T cells correlated inversely with declined FEV1 in COPD. CSE promoted the proliferation and inhibited the apoptosis of CD8^+^ T cells, while facilitated both the proliferation and apoptosis of CD8^+^ Tregs. Notably, the effects of CSE on CD8^+^ Tc/Tregs can be mostly simulated or attenuated by muscarine and atropine, the MR agonist and antagonist, respectively. However, neither muscarine nor atropine influenced the apoptosis of CD8^+^ Tregs.

**Conclusion:**

The results imply that cigarette smoking likely facilitates a proinflammatory state in smokers, which is partially mediated by MR dysfunction. The MR antagonist may be a beneficial drug candidate for cigarette smoke-induced chronic airway inflammation.

## Introduction

Cigarette smoke-induced pulmonary inflammation, such as chronic obstructive pulmonary disease (COPD), is the fourth leading cause of death worldwide [1–3]. Many lines of evidence indicate that adaptive immunity plays important roles in the development of COPD, and the systemic inflammation may be a result of overspill of inflammatory mediators from the lungs [4]. However, the immune regulation in smoking related airway inflammation has not yet been fully illuminated.

Previous studies have shown that CD8^+^ T cells in both lung parenchyma and pulmonary arteries are inversely correlated with pulmonary function [5], which suggest that CD8^+^ T cells (Cytotoxic T cells, Tc) may play a critical role in the pathogenesis of smoking related pulmonary inflammation. While much attention has been given to the subsets of CD4^+^ regulatory T cells (Tregs) for their role in the maintenance of immune homeostasis, recent findings have demonstrated that CD8^+^ Tregs display immune regulation functions as well [6,7]. Meanwhile, our previous study indicated that there was an inverse correlation between the circulating CD8^+^ Tregs and smoking index [8]. However, the precise role of CD8+ Tc/Treg cells in the pathogenesis of chronic airway inflammation remains unclear. Although some in vitro research has indicated that soluble components extracted from cigarette smoke can significantly reduce the proliferation and activation of T cell [9,10], the influence of cigarette smoke on CD8^+^ T cells remains unclear.

T cells express cholinergic receptors, including muscarinic acetylcholine receptors (mAChRs or MRs) and nicotinic acetylcholine receptors (nAChRs), and represent a cellular source of Ach [11]. In particular, our published data [12] and that of others [13] have reported that MR expression on human peripheral blood T cells (PBT cells) was mostly restricted to MR3, MR4 and MR5. COPD is a type of chronic inflammatory disease characterized by the hyperfunctioning of the cholinergic system [14] coupled with increased MR3 expression and CD8^+^-ACh binding on PBT cells [15], indicating that MRs play important regulatory roles in CD8^+^ T cell survival. In addition, our previous study indicated that the MR antagonist tiotropium bromide increased the number of CD8^+^ Tregs in cigarette smoke-induced COPD patients [16,17]. We therefore hypothesized that the cholinergic system may be involved in the pathogenesis of cigarette smoke-induced chronic inflammation by regulating T cells, particularly the CD8^+^ Tc/Tregs subsets.

In the present study, we attempted to investigate the effects of cigarette smoke extract (CSE) and MR signaling on the proliferation and apoptosis of CD8^+^ T cells and CD8^+^ Tregs. Our current data showed that cigarette smoking facilitates the formation of pro-inflammatory milieu and the development of COPD by causing an imbalance between CD8^+^ T and Treg cells, which is skewed toward CD8^+^ T cell-dominant phenotype. To the best of our knowledge, this is the first report showing that cigarette smoke may disturb the survival of CD8^+^ Tc/Tregs partially through MRs.

## Materials and Methods

### Subjects and sample collection

This study was approved by the Ethics Committee of Union Hospital, Tongji Medical School, Huazhong University of Science and Technology, and informed written consent was obtained from each donor. Based on the criteria supplied by the Global Initiative for Chronic Obstructive Lung Disease (GOLD) guidelines [18], we recruited patients with stable COPD (n = 18) who were free of exacerbation for at least 4 weeks at the time of blood draw. Meanwhile, healthy smokers (HS, n = 15) and healthy nonsmoker controls (HC, n = 15) with normal lung functions were also collected and were free of any lung or systemic disease. COPD patients and healthy smokers had a smoking history of 10 pack years or more. Exclusion criteria for the study included the following characteristics: other respiratory diseases apart from COPD, systemic autoimmune diseases, and treatment with anticholinergics, glucocorticoids or immunomodulators within 4 weeks prior the research. Peripheral blood samples were collected in heparin-treated tubes. As mentioned before [12], the peripheral blood T cells (PBT cells) were harvested and resuspended in complete RPMI 1640 medium plus 10% fetal bovine serum (FBS) and placed in an incubator at 37°C in 5% CO_2_ for subsequent experiments, depending on the availability of cells.

### Flow cytometric analysis

**Fig 1:**
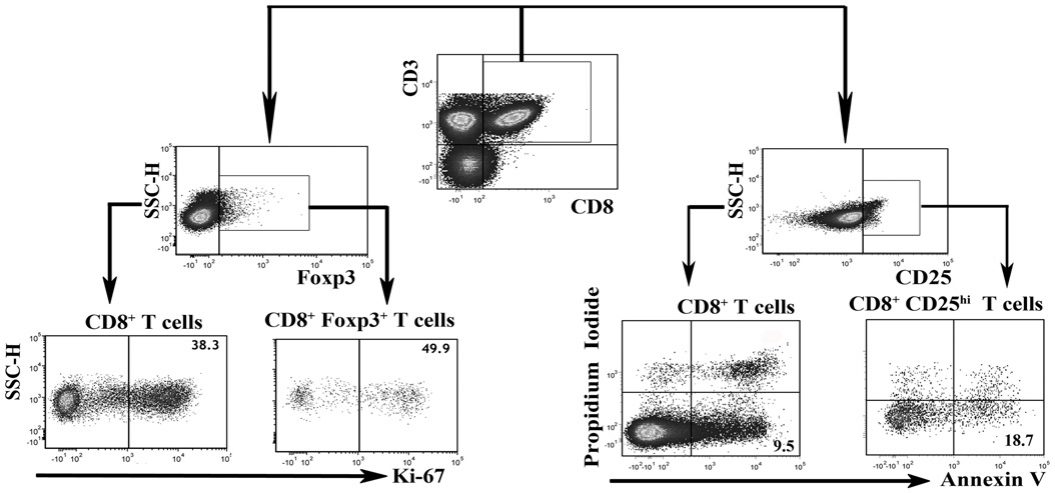
Representative dot plots show the gating strategy of the assays. In the proliferation assays, CD8^+^ Tregs were gated from CD8^+^ T cells (CD3^+^CD8^+^) based on their positive expression of Foxp3, and the expression of Ki-67 by CD8^+^ T cells and CD8^+^ Tregs was then further analyzed. However, in the apoptosis assays, CD8^+^ Tregs were gated from CD8^+^ T cells (CD3^+^CD8^+^) based on their high expression of CD25, and the apoptosis of CD8^+^ T cells and CD8^+^ Tregs gated on propidium iodide-negative and Annexin V-positive cells was then further analyzed.

The expression of surface marker and transcription factors by T cells was assessed by flow cytometry as previously described [12] after surface or intracellular staining with peridinin chlorophyll protein (PerCP)-Cy5.5–conjugated anti-CD3 (eBioscience, San Diego, CA, USA), fluorescein isothiocyanate (FITC)-conjugated anti-CD8 (BD Biosciences, San Jose, CA, USA), phycoerythrin (PE)-Cy7-conjugated anti-CD25 (BD Biosciences), and PE-conjugated anti-Foxp3 (eBioscience) antibodies. Isotype controls were generated to perform compensation and to confirm antibody specificity. Flow cytometry was performed on a FACS Canto II (BD Biosciences) and analyzed using BD FCSDiva Software and FCS Express 4 software (De Novo Software, Los Angeles, CA, USA). Fig 1 showed the gating strategy.

### CSE preparation

CSE was prepared using the method of Blue and Janoff [19]. The CSE concentrations used in the experiments were chosen in consistent with our previous report [12]. Briefly, cigarette smoke was drawn into a 50 ml plastic syringe, and smoke was then slowly bubbled into a tube containing 5 ml of sterile RPMI 1640 medium. A total of 5 ml of CSE was produced from two cigarettes (Huang Helou, Wuhan, Hubei, China). Next, the CSE solution was filtered through 0.22-μm filters. To ensure the vitality of the substances in the CSE, all of the CSE was prepared within a half hour before each experiment.

### Proliferation and apoptosis of T cells

Proliferation and apoptosis assays in vitro were performed as described in detail previously [12]. In brief, PBT cells were maintained in the presence of CSE, MR agonist muscarine (Mus, 50 μM, Sigma-Aldrich, St. Louis, MO, USA), and MR antagonist atropine (Atro, 100 μM, Sigma-Aldrich), either alone or in various combinations. In the proliferation assays, 1 μg/ml PHA and 50 ng/ml PMA (both from Sigma-Aldrich) were added to the medium to stimulate T cell proliferation. After 5 days, the cells were harvested and the proportions of proliferating and apoptotic T cells were determined by flow cytometry with Alexa Fluor^®^ 647-conjugated anti-human Ki-67 (eBioscience), allophycocyanin (APC)-conjugated Annexin V and propidium iodide (PI) (Annexin V Apoptosis Detection Kit APC; eBioscience).

### Statistical analysis

Data are expressed as the mean ± SEM (unless otherwise indicated). Multiple comparisons between different groups were performed using the nonparametric Kruskal-Wallis test followed by Dunn’s post hoc test. Correlations between variables were determined using the Spearman rank test. Data analysis was performed using GraphPad Prism v.5.01 software (GraphPad Software, La Jolla, CA, USA), and two-tailed P values of less than 0.05 were considered statistically significant.

## Results

### Subject Demographics

The main clinical characteristics of the three groups studied are presented in Table 1. The unequal sex ratio was consistent with the much higher prevalence of COPD in males than in females. The smoking history of smokers with normal lung function and patients with COPD was similar. Patients with COPD showed moderate airflow obstruction, whereas the other two groups have normal spirometry.

**Table 1:** Demographics and clinical characteristics of all participants.

Variables	HC	HS	COPD
Subjects (No.)	15	15	18
Age (year)	54.9 ± 2.4	59.2 ± 1.3	58.3 ± 1.9
Gender (Male/Female)	13/2	14/1	16/2
Tobacco (pack-year)	-	39 (12–64)	38 (14–70)
FEV1 (% predicted)	94.0 ± 0.8	92.3 ± 0.6	50.1 ± 2.3*^#^
FEV1/ FVC (%)	81.38 ± 1.72	79.60 ± 1.52	50.83 ± 1.85*^#^

The data are represented as the mean ± SEM or median (range). FEV1: forced expiratory volume in one second; FVC: forced vital capacity.

* P < 0.05 vs. the HC group;

^#^ P < 0.05 vs. the HS group.

### Elevated percentages of CD8^+^ T cells correlated inversely with declined FEV1 in COPD

**Fig 2:**
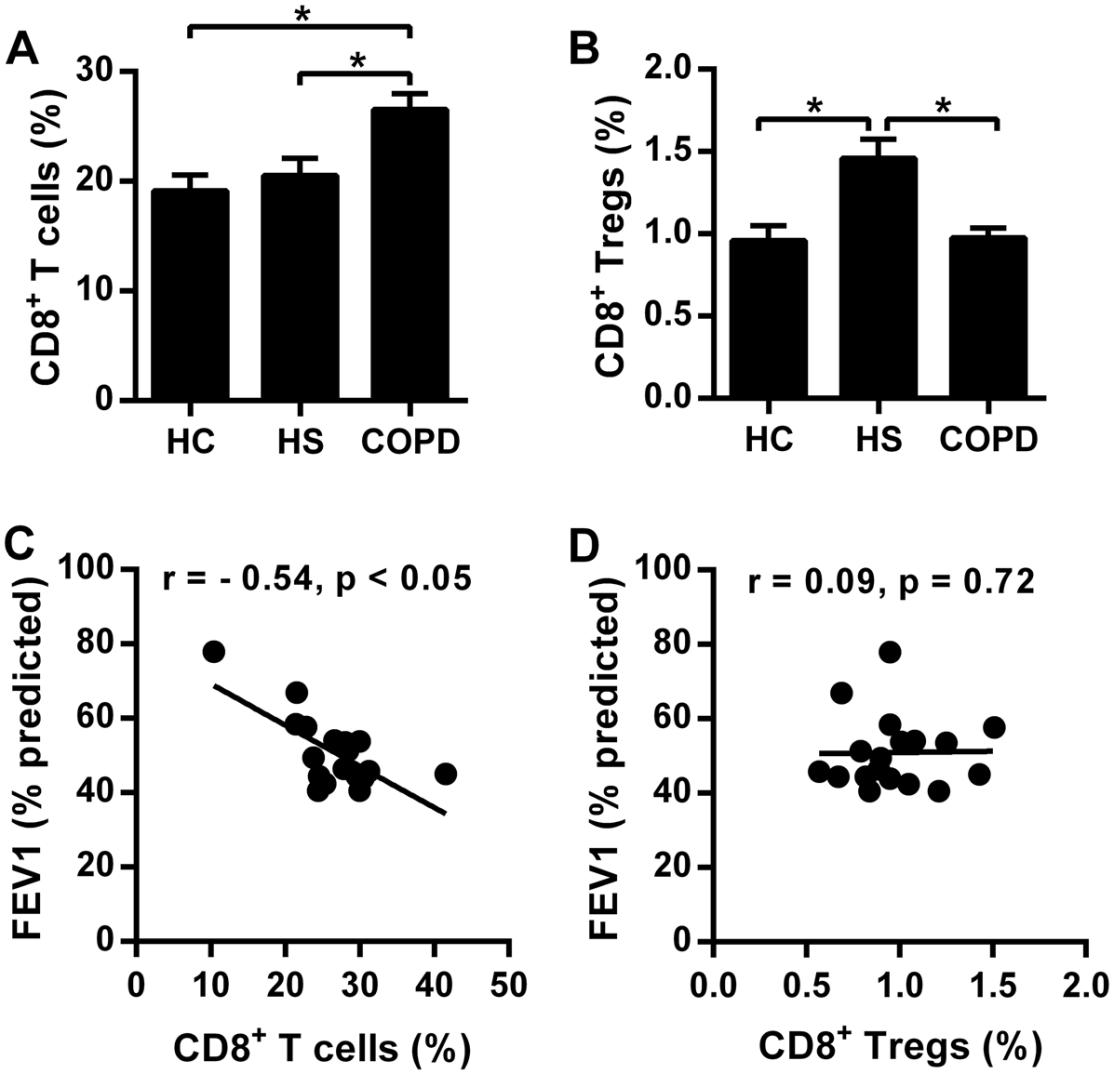
Imbalance of circulating CD8^+^ Tc/Tregs in patients with chronic obstructive pulmonary disease (COPD). Comparisons of percentages of CD8^+^ T cells (A) and CD8^+^ Tregs (B) in peripheral blood from healthy controls (HC, n = 15), healthy smokers (HS, n = 15) and COPD patients (n = 18). Correlation of CD8^+^ T cells (C) and CD8^+^ Tregs (D) with FEV1% predicted value in COPD patients (n = 18). * P<0.05.

We first investigated the frequencies of CD8^+^ Tc/Treg cells in peripheral blood of different groups. The percentage of CD8^+^ T cells was significantly higher in patients with COPD (mean = 26.54%) than in HS (mean = 20.53%, p < 0.05) or HC (mean = 19.11%, p < 0.05) subjects. The levels of CD8^+^ T cells in HS subjects were slightly higher than those in the HC subjects, although the difference did not reach statistical significance (Fig 2A). Furthermore, the frequency of CD8^+^ Tregs was markedly higher in HS donors (mean 1.46%) than in COPD patients (mean = 0.98%, p < 0.05) and HC groups (mean = 0.96%, p < 0.05) (Fig 2B).

Single regression analysis between lymphocytes and airflow obstruction in COPD patients was performed. We observed that the levels of CD8^+^ T cells displayed a negative correlation with FEV1 (% predicted) (r = - 0.54, p < 0.05) (Fig 2C), supporting a possible role for CD8^+^ T cells in the airway inflammation. However, frequency of CD8^+^ Tregs did not correlate with pulmonary function tests (r = 0.09, p = 0.72) (Fig 2D).

### Impacts of CSE and muscarinic receptors on CD8^+^ T cells survival

**Fig 3:**
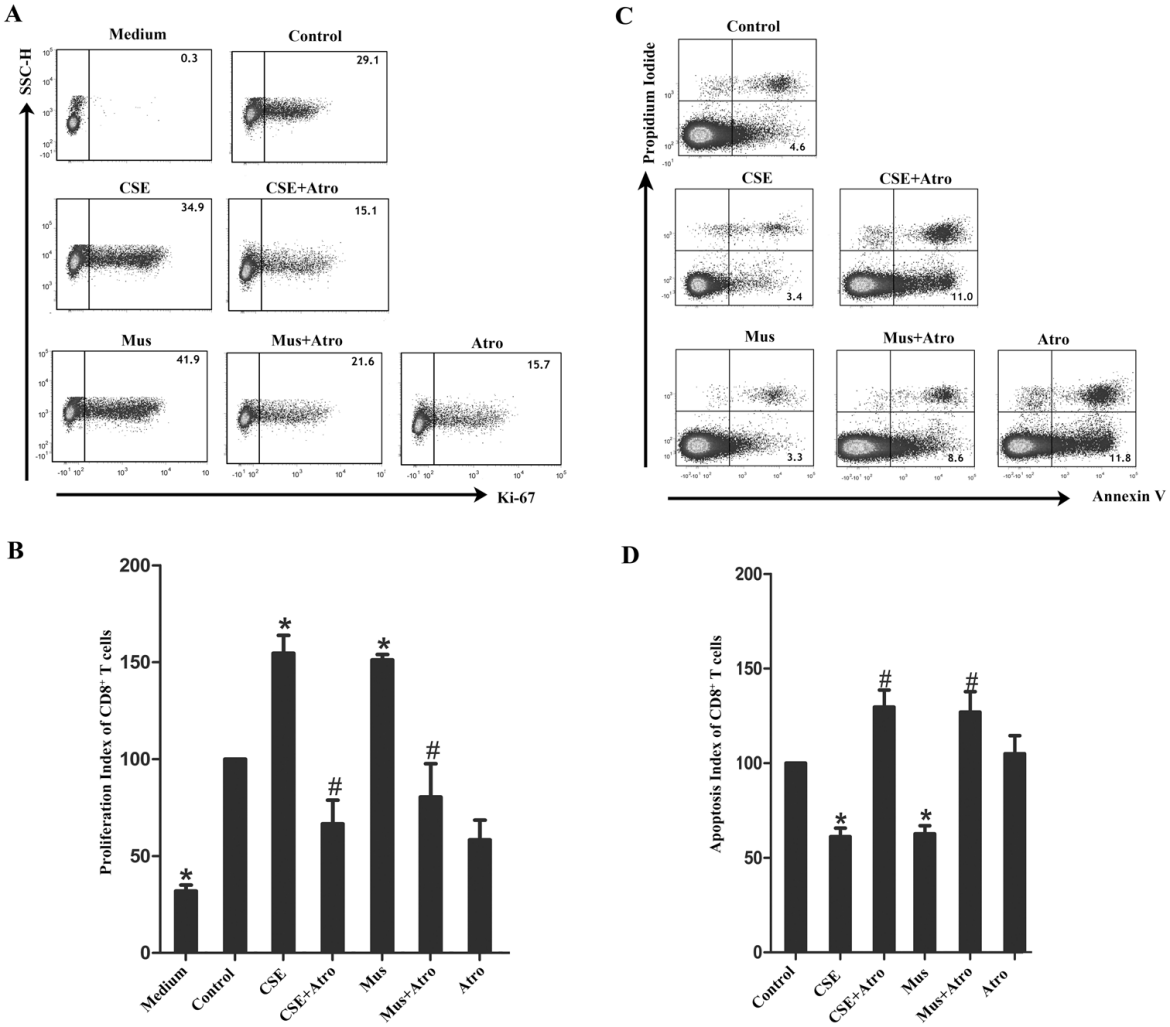
Effects of CSE and muscarinic receptors on proliferation and apoptosis of CD8^+^ T cells. PBT cells from healthy nonsmokers were cultured for 5 days in the presence of CSE and MRs agonist/antagonist, either alone or in various combinations. (A) In the proliferation assays, medium combined with PHA and PMA was considered as control treated wells. Ki-67^+^CD8^+^ T cells (excluding CD8^+^ Tregs) were examined by flow cytometry, and the representative flow cytometric dot plots are shown. (B) The proliferation index of control wells was considered to be 100, and data are expressed as fold increase relative that in the control wells (n = 5). (C) In the apoptosis assays, medium alone was considered as control treated wells. Apoptotic CD8^+^ T cells (excluding CD8^+^ Tregs) were examined by flow cytometry, and the representative flow cytometric dot plots are shown. (D) The apoptosis index of control wells was considered to be 100, and data are expressed as fold increase relative that in the control wells (n = 6). The results are reported as the mean ± SEM. *P<0.05 compared with the control wells; #P<0.05 indicates CSE plus Atro compared with CSE or Mus plus Atro compared with Mus.

To investigate the effects of cigarette smoke on the proliferation and apoptosis of CD8^+^ T cells (excluding CD8^+^ Tregs), we performed proliferation and apoptosis assays using PBT cells obtained from healthy nonsmokers. As shown in Fig 3A and 3B, Ki-67^+^ CD8^+^ T cells were significantly more abundant in CSE and Mus treated group, which can be abolished by Atro, a MR antagonist.

Additionally, CSE reduced the apoptosis of CD8^+^ T cells (excluding CD8^+^ Tregs), whereas Atro treatment completely abrogated this anti-apoptotic effect. Indeed, both CSE and Mus reduced the apoptosis of CD8^+^ T cells, which can be fully aboished by Atro (Fig 3C and 3D).

### Impacts of CSE and muscarinic receptors on CD8^+^ Tregs survival

We further analyzed the effects of cigarette smoke on CD8^+^ Treg. The proliferation of CD8^+^ Tregs was analyzed using CD3^+^CD8^+^Foxp3^+^ as the Treg phenotypic marker set (Fig 1). Considering that the cell membrane must be kept intact for the detection of apoptotic cells by PI and Annexin V (thus, Foxp3 staining cannot be achieved), CD3^+^CD8^+^CD25^hi^ was used as the CD8^+^ Treg phenotypic marker profile (Fig 1). As expected, the percentages of CD8^+^CD25^hi^ T cell subset overlapped with CD8^+^FoxP3^+^ cells (data not shown).

**Fig 4:**
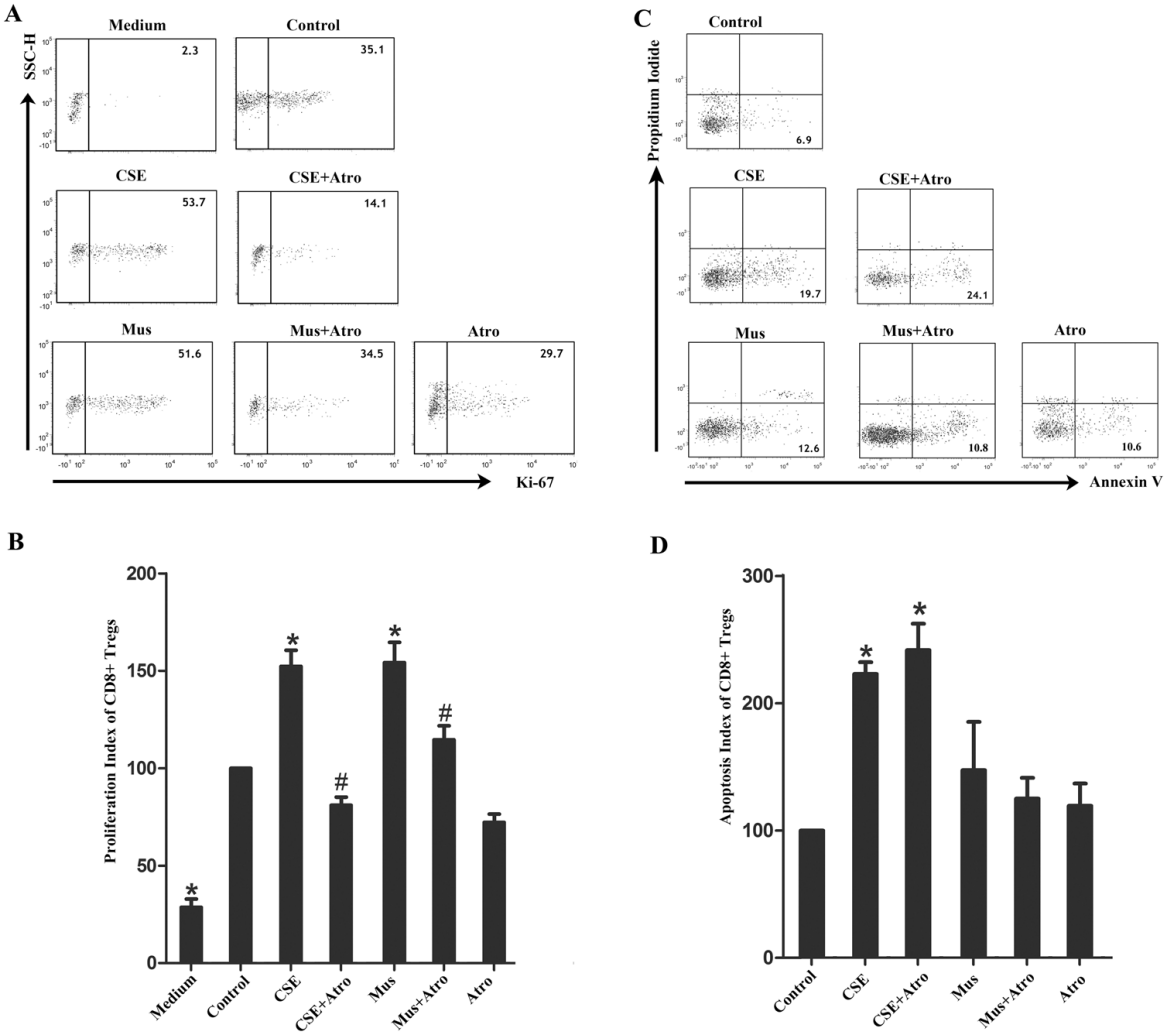
Effects of CSE and muscarinic receptors on proliferation and apoptosis of CD8^+^ Treg cells. PBT cells from healthy nonsmokers were cultured for 5 days in the presence of CSE and MRs agonist/antagonist, either alone or in various combinations. (A) In the proliferation assays, medium combined with PHA and PMA was considered as control treated wells. Ki-67^+^CD8^+^ Treg cells were examined by flow cytometry, and the representative flow cytometric dot plots are shown. (B) The proliferation index of control wells was considered to be 100, and data are expressed as fold increase relative that in the control wells (n = 5). (C) In the apoptosis assays, medium alone was considered as control treated wells. Apoptotic CD8^+^ Treg cells were examined by flow cytometry, and the representative flow cytometric dot plots are shown. (D) The apoptosis index of control wells was considered to be 100, and data are expressed as fold increase relative that in the control wells (n = 6). The results are reported as the mean ± SEM. *P<0.05 compared with the control wells; #P<0.05 indicates CSE plus Atro compared with CSE or Mus plus Atro compared with Mus.

As indicated in Fig 4A and 4B, for CD8^+^ Tregs from healthy nonsmokers, both CSE and Mus significantly facilitated proliferation, which can be blocked by Atro. Unexpectedly, CSE robustly promoted CD8^+^ Treg apoptosis in healthy nonsmokers (Fig 4C and 4D). However, neither MR agonist nor antagonist influenced apoptosis, indicating that CD8^+^ Treg apoptosis was less sensitive to MR modulation.

## Discussion

Previous studies have mainly focused on bronchodilation of MR-based treatments for chronic airway inflammation. However, the potential for anti-inflammatory benefits in addition to bronchodilation is not well understood. To the best of our knowledge, the present study shows for the first time that cigarette smoke disturb the survival of CD8^+^ T/CD8^+^ Tregs partially through MRs-dependent mechanisms.

Many studies have reported increased percentages of CD8^+^ T cells confined to the lung of COPD patients, such as pulmonary parenchyma and lung arteries [5], central and peripheral airways [20,21], and bronchoalveolar lavage (BAL) [22]. Remarkably, our findings extend these observations by showing that the number of CD8^+^ T cells is also increased in the peripheral blood of COPD patients. The presented results agree with a previous study in blood [15], suggesting that the origin of the abnormal CD8^+^ T cell infiltration seen in the lungs of COPD patients is a systemic rather than a local event. In support of these findings [5,20], we have demonstrated that the increased frequency of circulating CD8^+^ T cells in patients with COPD shows an inverse correlation with the declined lung function. There is growing interest in the potential mechanisms by which CD8^+^ T cells worsen lung function in cigarette smoke-induced COPD. Hamid and his coworkers demonstrated that exposure of CSE lead to the activation of CD8^+^ T cells, resulting in the production of IL-1β, IL-6, IL-10, IL-12p70, TNF-α and IFN-γ, which was associated Toll-like receptor (TLR)-4 and TLR9 [23]. And, in keeping with these findings, the lung function of COPD patients was inversely associated with the percentage of BAL and intraepithelial CD8^+^ T cells producing TNF-α [24], as well as the frequency of CD8^+^ T cells activation and CD8 IFN-γ production [25]. We could speculate this increased cytokines can cause the aggregation of other inflammatory cells to the lung, ultimately perpetuating the inflammation and damage observed in COPD patients, even in individuals who have quitted smoking. Obviously, much work remains to be done before our understanding of emphysema pathogenesis.

Interestingly, we showed that cigarette smoking increased the number of circulating CD8^+^ Tregs in healthy smokers, but not in somkers with COPD. The increased CD8^+^ Foxp3^+^ Tregs observed in healthy smokers is in agreement with the results of previous report [26], which revealed a prominent upregulation of CD8^+^ γδ^+^ Tregs in bronchoalveolar lavage from smokers with normal lung function. Given the relevance of Foxp3^+^ and γδ^+^ T-lymphocytes in tissue homeostasis, these observations are compatible with a physiological response aimed to protect the lungs from the injury caused by cigarette smoking.

According to genetic knockout experiments, the cholinergic pathway is involved in the regulation of CD8^+^ T cells [27], and increased ACh-binding to CD3^+^CD8^+^ T cells in the peripheral blood occurs in COPD patients [15]. However, the exact effects of MRs on CD8^+^ T cells during the development of COPD remain unknown. In this present study, cells were incubated with CSE or Mus (with or without Atro) to discern whether smoking and MRs affected the survival of CD8+ T cells by using the proliferation assay. Our present data indicated that both CSE and Mus significantly promoted the proliferation of CD8^+^ T cells from healthy nonsmokers. Our data are consistent with previous research showing that MR activation could promote CD8^+^ cytolytic T cell generation [27]. Considering that CSE could promote the proliferation of human fetal lung fibroblasts by increasing MR1 and MR3 expression and ERK1/2 and NF-κB activation [2], combing with our finding that MR antagonist Atro could abrogate the pro-proliferative effect of CSE, we presumed that the effect of CSE on CD8^+^ T cells may function through MRs. Our study is the first to show that CSE could promote CD8^+^ T cell proliferation by activating the muscarine system, thereby contributing to the increased numbers of CD8^+^ T cells in the airways of COPD patients.

CSE may exert effects on apoptosis of CD8^+^ T cells through a variety of substances, as cigarette smoke comprises more than 4,000 components, including endotoxin, reactive oxygen species and free radicals [3]. We showed that CSE and Mus can promote apoptosis of CD8^+^ T cells, which can be completely abrogated by Atro, indicating that the anti-apoptotic role of CSE is MR dependent. Our results were consistent with the previous study showing that the apoptosis of CD8^+^ T cells in the peripheral blood of COPD patients can be induced by anticholinergic drugs in a caspases3/8 and IκB-dependent manner [15].

Notably, we also have shown for the first time that CSE paradoxically played both pro-apoptotic and pro-proliferative roles in CD8^+^ Tregs. Thus, the actual in vivo effect of cigarette smoking on CD8^+^ Tc/Tregs is linked to population susceptibility: in smokers with normal lung function, pro-proliferative role on CD8^+^ Tregs is in a dominant position, which successfully delays the inflammatory cells (including CD8^+^ and CD4^+^ lymphocytes) amplification and protects the lungs from the injury caused by current tobacco smoking; In smokers with COPD, however, pro-apoptotic role on CD8^+^ Tregs takes precedence, and chronic cigarette smoke exposure in COPD patients results in sustained cholinergic signaling activation and CD8^+^ T cell infiltration, eventually impairing the immunity of COPD patients.

We speculate that certain master switch controls the different effects of CSE on proliferative and apoptotic level of CD8^+^ Treg cells. On the one hand, it is reported that the effects of CSE on T cells development depend on inflammatory milieu and differentiation condition [28]. It is also known that the cytokine milieu of COPD differs from that of healthy controls, with IL-12 being the predominant cytokine and IL-4 being the deficient cytokine [29,30]. Therefore, the difference in the inflammatory milieu could trigger the dichotomous effects of CSE treatment on healthy- vs COPD- smokers. On the other hand, a deficiency of α-1-antitrypsin (α-1AT) is the only known gene linked to the early onset of emphysema, and there is a large phenotypic variability even among carriers of different forms of α-1AT proteins [31,32]. In particular, scanning the entire genome for SNPs has been used to identify several new risk genes involved in human COPD, such as the α- nAChR (CHRNA 3/5) [33]. These findings further indicate that genetic susceptibility may be the master switch that controls different proliferative and apoptotic level. Predictably, clarifying distinct clinical phenotypes and identifying the susceptible genotypes will not only provide personalized treatment to smoke-induced emphysema, but also will provide methods to predict smokers who are at a higher risk of developing COPD.

Additionally, although MR agonist had pro-proliferative effect on CD8^+^ Tregs resembling that of CSE, MRs did not seem to affect CD8^+^ Treg apoptosis, indicating that CSE promotes the apoptosis of CD8^+^ Treg cells in a MR-independent signaling pathway: Firstly, since we have previously reported that the expression of anti-inflammatory α7 nAChR by circulating CD4^+^ T cells was reduced in smokers with COPD [12], CSE may disrupt a signaling pathway of nicotinic but not muscarinic acetylcholine receptor, contributing to the apoptosis of CD8^+^ Tregs in susceptible populations. Secondly, as cigarette smoke is a potent source of oxidative stress and apoptosis for human lung fibroblasts (HFL-1) [34], CSE may similarly contribute to the apoptosis of CD8^+^ Treg cells by the pathway of oxidative stress. Lastly, numerous substances besides nicotine were contained in CSE, so further works will be necessary to clarify the mechanisms of action of CSE in vivo.

## Conclusions

Our experiments demonstrated that cigarette smoke may directly cause an imbalance of pro- and anti-inflammatory response via its effects on CD8^+^ T cell/CD8^+^ Treg survival, which may explain the sustained impairment of immune defenses in COPD patients who continue to smoke. Furthermore, MR activation is involved in the effect of CSE on CD8+ T cells’ proliferation/apoptosis and CD8+ Tregs’ proliferation. Thus, targets on MRs-dependent mechanisms may abrogate the effects of cigarette smoke to some extent and thereby regulate abnormal activation of the inflammatory response. These findings raise the possibility that MR antagonists may restore a balance of pro-/anti-inflammation, which are therapeutically beneficial in addition to bronchodilation for chronic airway inflammation.

## Supporting Information

S1 File The underlying data points for the graphs reported in the figures. (ZIP)
